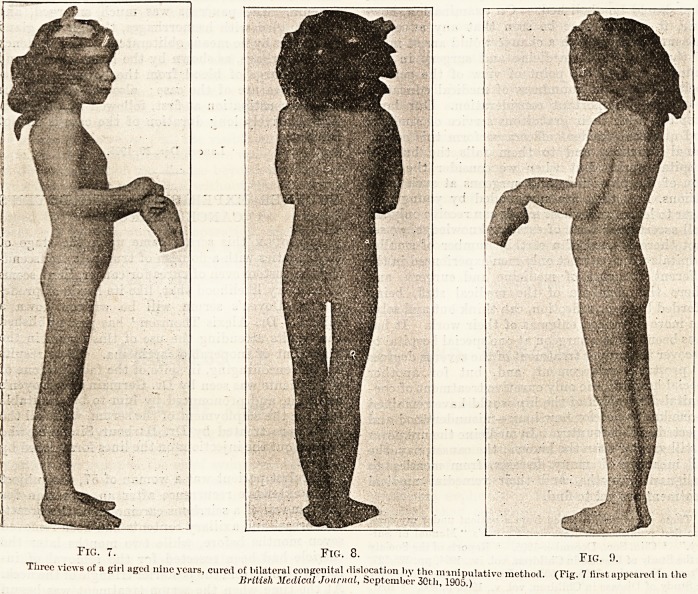# The Modern Treatment of Congenital Dislocation of the Hip-Joint

**Published:** 1906-01-20

**Authors:** J. Jackson Clarke

**Affiliations:** Surgeon to the North-West London Hospital, and to the City of London Orthopædic Hospital


### Hospital Clinics.

### THE MODERN TREATMENT OF CONGENITAL DISLOCATION OF THE HIP-JOINT.

By J. Jackson Clarke, M.B.Lond., F.R.C.S., Surgeon to the North-West London Hospital, and to the City of London Orthopædic Hospital.

Until the year that has just passed away congenital dislocation of the hip-joint, at once the commonest and most formidable of congenital dislocations, was counted in this country as an incurable condition. How great a disability is even a unilateral dislocation only the patient and the patient's parents can tell. Those surgeons who have been in the habit of seeing a considerable number of these patients for many years together also know how severely handicapped they are in the race of life. Patients with double dislocations are, as a rule, even more distressingly crippled. A child in this condition is cut off from most games and exercises that give pleasure and tend to promote health and strength; he is precluded from following any pursuit requiring rapid locomotion, and hence from earning a livelihood, except in one of the sedentary and usually overstocked occupations. Only in very rare cases the head of the femur is held naturally in a favourable position so that the patient is able to walk fairly well and is free from those secondary spinal deformities: lordosis and lateral curvature, that so frequently supervene on congenital dislocation.

A faint idea of the discomfort that a patient suffers in case of double dislocation may be seen in Figs. 1, 2, and 3.[1] Such illustrations, after all, show but the deformity produced, and not the burdensome limitations of function. One of the latter is indicated in Fig. 5, taken from a boy aged 6¾ years on whom I operated recently for double dislocation. Figs. 4 and 6 show the beginnings of the lordosis, which has become more pronounced in the older patient, Figs. 2 and 3. Fig. 5 shows the boy's knees separated to their fullest extent—*i.e.* instead of the degree of abduction of each thigh being about 85 degrees it is barely 30. We may compare with the above the appearance of a girl who had a condition quite as bad as that shown in the boy, and who, if she had been left untreated, would have been nearly as old and as severely deformed as the girl in Figs. 1-3. It is now three years since she was operated on by the manipulative method of Lorenz, and she is now perfect in form, and she has a full physiological range of movement in both the hip-joints, the heads of which are firmly established in the acetabula. This patient has been completely cured of what till recently was, and by many surgeons is even now, regarded as an incurable condition. Since this patient was operated on for me by Lorenz I have published [2] two series, each of 10 successive cases, the results of which I may briefly state here.

Fig. 1.

Fig. 2.

Fig. 3.

Three views of a girl aged fifteen years; a typical instance of bilateral congenital hip-dislocation.

In the first series the ages of the patients varied from three to nine years, and two of them were cases of bilateral dislocation; thus there were 12 joints. Of these nine are cured. In three the head of the femur was "anteverted," as it is termed, at the operation. Of these anteversions one has relapsed, one is doubtful, the third is firmly established, the head of the femur being placed beneath the anterior superior spine, and the new joint having a full physiological range of movement. This patient is a girl aged eight years, who had bilateral dislocation. She now walks as firmly with this "anteverted" as with the other joint which is completely cured. In the second series, also of ten cases (ages of patients being from two to nine years), there were three double dislocations. Complete anatomical reduction has been effected in every one of these 13 joints. There was no kind of operative mishap in either series. The magnitude and importance of this advance in surgical knowledge can only be realised by comparing the opinions of surgeons of experience before they had come to understand the scientific basis and the technical details of the manipulative operation of Lorenz, with their feelings after they had realised that a new field of successful surgery had really been won by Lorenz's splendid piece of progressive scientific labour, which began in 1892 with his first open operation, and was finished in 1896 when he obtained his first complete success by his manipulative method, and thereupon relinquished the former operation once for all. This aspect of the matter may best be illustrated by comparing the opinion expressed by Mr. A. H. Tubby as recently as 1904 with that held by the same surgeon at the present time. Speaking at Bristol [3] on June 18, 1904, he observed:—"The so-called 'bloodless method' was not entirely Lorenz's. It was a modification of a method which was brought forward by Paci in Pisa in 1894, the only difference being that Lorenz is more violent than Paci. Those who knew the literature of the subject would not assign the entire credit to Lorenz for the operation. . . . Mr. Tubby had done the operation several times, and his own impression was that the operation should not be vaunted as a cure, and where anything like excessive force was called for it was the surgeon's duty to desist, because when force was called for it was almost certain to result in a bad dislocation or an untoward accident." A little over a year later the same surgeon has written [4]:—"A good deal of controversy has arisen on the question of the priority of reduction of congenital dislocation of the hip by manipulation. Those who would belittle Lorenz's work, give to Paci of Pisa the credit

FIG. 4. FIG. 5. FIG. 6.

Three views of a boy aged six years and nine months, the subject of bilateral dislocation. Fig. 5 shows the fullest extent to which patient could abduct the legs.

of originating the operation, and are of opinion that Lorenz has added little to Paci's work. But this attitude arises from a confusion in the minds of those who criticise the methods of the two surgeons. The Lorenz reduction is accomplished by the ultra-physiological abduction, the head entering the acetabulum over its posterior rim; whereas the Paci movements bring the head in over the inferior part of the acetabulum. . . . The result of the past two years' work seems to show that, in the future, the irreducible cases will gradually disappear to a vanishing point." In the interval between these factory that both surgeons relinquished the method, reverting to the open operation that Lorenz, after an unrivalled experience of it, had definitely abandoned in favour of his manipulative method. My own results with the latter had, up to the year 1903, been as unsatisfactory as those of Burghard and Openshaw, only an occasional success being obtained. This had led me to try the open operation in one instance, and it might have led me to abandon manipulative methods had I not been convinced by dissections I made some 15 years ago in a case of double congenital dislocation that this deformity

FIG. 7.

FIG. 8.

FIG. 9.

Three views of a girl aged nine years, cured of bilateral congenital dislocation by the manipulative method. (Fig. 7 first appeared in the *British Medical Journal*, September 30th, 1905.)

two statements I had shown cases and read a paper [5] in which I detailed the practical points in Lorenz's manipulative operation and the after-treatment.

A just appreciation of the value of Lorenz's manipulative method of treating congenital dislocation of the hip can only be arrived at by the study of the results obtained in a considerable number of consecutive cases, the patients being within the prescribed limits of age—*i.e.* up to eight years in bilateral and up to ten years in unilateral cases. Before the publication of my two series the results of only two such series of cases had been published in this country by Burghard [6] and Openshaw [7]: the former had one success in 10, the latter three "good results" in 20 cases. These results were so unsatis- ought to be curable by manipulation if the right way could only be found. Lorenz's visit to this country in 1903, and his patient demonstration of every technical detail of his method enabled me to see wherein lay the sources of failure, and also the elements of danger, all of which can be completely avoided by experience. Thus, looking back, one realises how very narrowly we in this country escaped an indefinite delay in accepting a really great advance in surgery—one that has rescued a grievous disability from a position of hopelessness to one of success: from the category of incurable, to that of completely and safely curable affections. There is but one advance in surgery that has been effected within the last 20 years that is of as great magnitude as Lorenz's treatment of congenital dislocation of the hip. This is Freyer's operation for enucleation of the hypertrophied prostate gland—an operation that has already saved an incalculable amount of human suffering and prolonged many useful lives. At the present time, when there are to be solved so many urgent problems as to hospitals and medical education, it is very necessary to view each problem from every aspect. The question of finance is as serious in the matter of hospital administration as it is in the political world. From the point of view of expense it might be thought that all special hospitals, save those for fevers and the like, should be abolished. On examination, however, it will at once be seen that any sweeping measure to effect such a change would arrest much of the progress of medicine and surgery in this country. From the point of view of the general social welfare the soundness of medical education is the most important consideration. Our large hospitals with their gratuitous service of students and qualified resident officers perform this educational function, and to them falls the bulk of hospital work. But when we consider the position of the physicians and surgeons at such institutions, how they are surrounded by young men eager to learn, and whose minds can receive only the well-ascertained facts of existing knowledge, we see that there is need of a certain number of smaller hospitals which attract only men experienced in the different branches of medicine and surgery, and where the members of the medical staff, being afforded time for reflection, can think out and solve the more advanced enigmas of their work. It has thus been left to a surgeon at one special hospital to discover the proper treatment of the severer degrees of prostatic enlargement, and, but for another special hospital, the only curative treatment of congenital dislocation of the hip would have remained—one knows not for how long—misunderstood and rejected in this country. In medicine the unknown is still greater than the known: the causes, nay, the full meaning of many diseases, from measles to malignant growths, and their remedial medical treatment are yet to find.

[1] These photographs, taken from a patient under my care, were first published in Rose and Carless's "Manual of Surgery." [2] Clin. Soc., December 1905. [3] Reports of the Society for the Study of Disease in Children, vol. iv., 1904, pp. 326-327. [4] Pract., November 1905, p. 683. [5] Reports of the Society for the Study of Disease in Children, vol. v., 1905, p. 290. [6] Brit. Med. Jour., October 19, 1901. [7] Trans. Clin. Soc., 1903, p. 146.

## Figures and Tables

**Fig. 1. Fig. 2. Fig. 3. f1:**
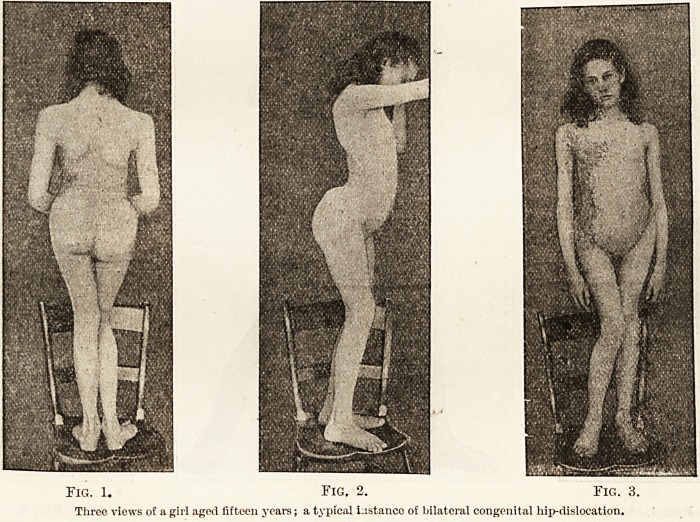


**Fig. 4. Fig. 5. Fig. 6. f2:**
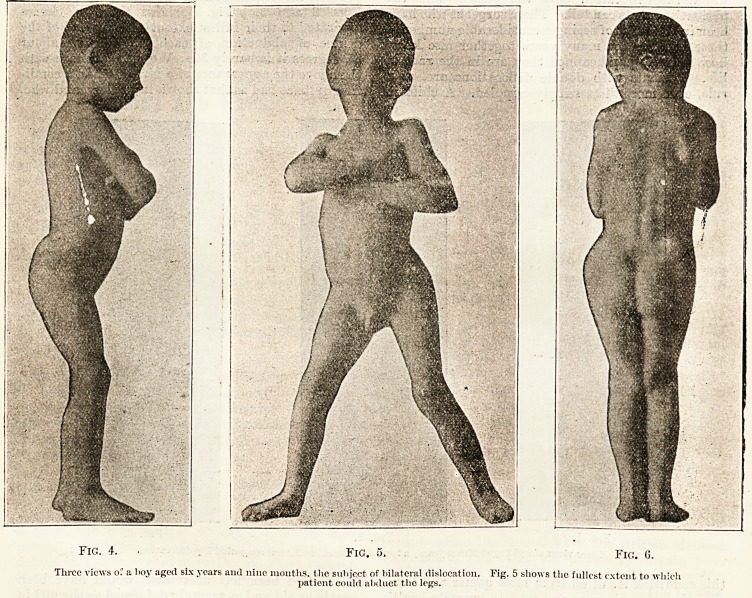


**Fig. 7. Fig. 8. Fig. 9. f3:**